# Navigating the Path Back: A Course for Medical Students Returning from Leave and Their Outcomes

**DOI:** 10.1007/s40670-025-02345-4

**Published:** 2025-03-04

**Authors:** Eric Nolan, Rakhi Gupta Basuray, Chirag A. Patel, Kimberly Tartaglia

**Affiliations:** 1 The Ohio State University College of Medicine, Columbus, OH 43210 USA; 2 Nationwide Children’s Hospital, Columbus, OH 43205 USA

**Keywords:** Undergraduate medical education, Medical student, Leave of absence, Clerkship

## Abstract

Leaves of absence (LOA) from medical school increase student stress and academic risk. We developed a course to support students returning to the clerkship year after a leave. The course was evaluated favorably by students and was reported to effectively prepare them for their clerkship year. We compared academic outcomes for course participants, students who took a LOA but did not participate in RICM, and traditional students. Planned LOA participants performed similarly to traditional students. However, students with unplanned LOAs faced higher academic risks and attrition, with course participation being associated with a reduction in post-clerkship delays in graduation.

**Supplementary Information:**

The online version contains supplementary material available at 10.1007/s40670-025-02345-4.

## Background

While graduation rates at US medical schools have remained stable over the last 25 years with approximately 97% of students graduating, 16% of students extend enrollment beyond the traditional 4 years [1]. At our medical college, approximately 12% of clerkship starters (“Year 3” students) are returning from a leave of absence (LOA) and another 10% delay graduation after starting their clerkship year. Reasons for a LOA prior to clerkship year range from pursuing additional degrees, research, academic decompression, and addressing personal or family concerns.

A LOA is associated with an increased risk of academic concerns and attrition during medical school [2]. However, risk varies by LOA reason, with higher attrition rates among students taking a LOA for reasons other than research or joint degrees [3]. The clerkship year marks a shift from foundational learning to clinical immersion, which can provoke stress and uncertainty about fitting into this new role [4]. While some reports suggest that factors such as institutional support, clear communication, and resources might help mitigate this stress, the evidence is limited, primarily based on a small survey of students’ experiences [5].

Although there are published reports on supporting medical students as they transition into the clerkship year [6–8], there is limited data on how to assist students returning from a LOA at this stage. Recognizing this gap, we developed the “Re-Introduction to Clinical Medicine” (RICM) course in 2018 to support students returning from a LOA as they begin their clerkship year. This 2-week program was designed to help students reintegrate into the medical curriculum. This report reflects on the course’s design and impact over 7 years and analyzes academic outcomes for participants.

## Activity

The RICM course provided a safe, un-graded space for applied learning and deliberate practice as students returned to the medical curriculum and clinical experiences. The course was offered in the 2 weeks before the start of the clerkship year to any student starting it directly after a LOA. The course revisited patient care and communication skills while developing clinical reasoning and applied medical knowledge. It included practical training, such as electronic medical record (EMR) navigation, along with direct observation and feedback. RICM combined small group classroom learning with an immersive inpatient internal medicine team experience.

**Fig. 1:**
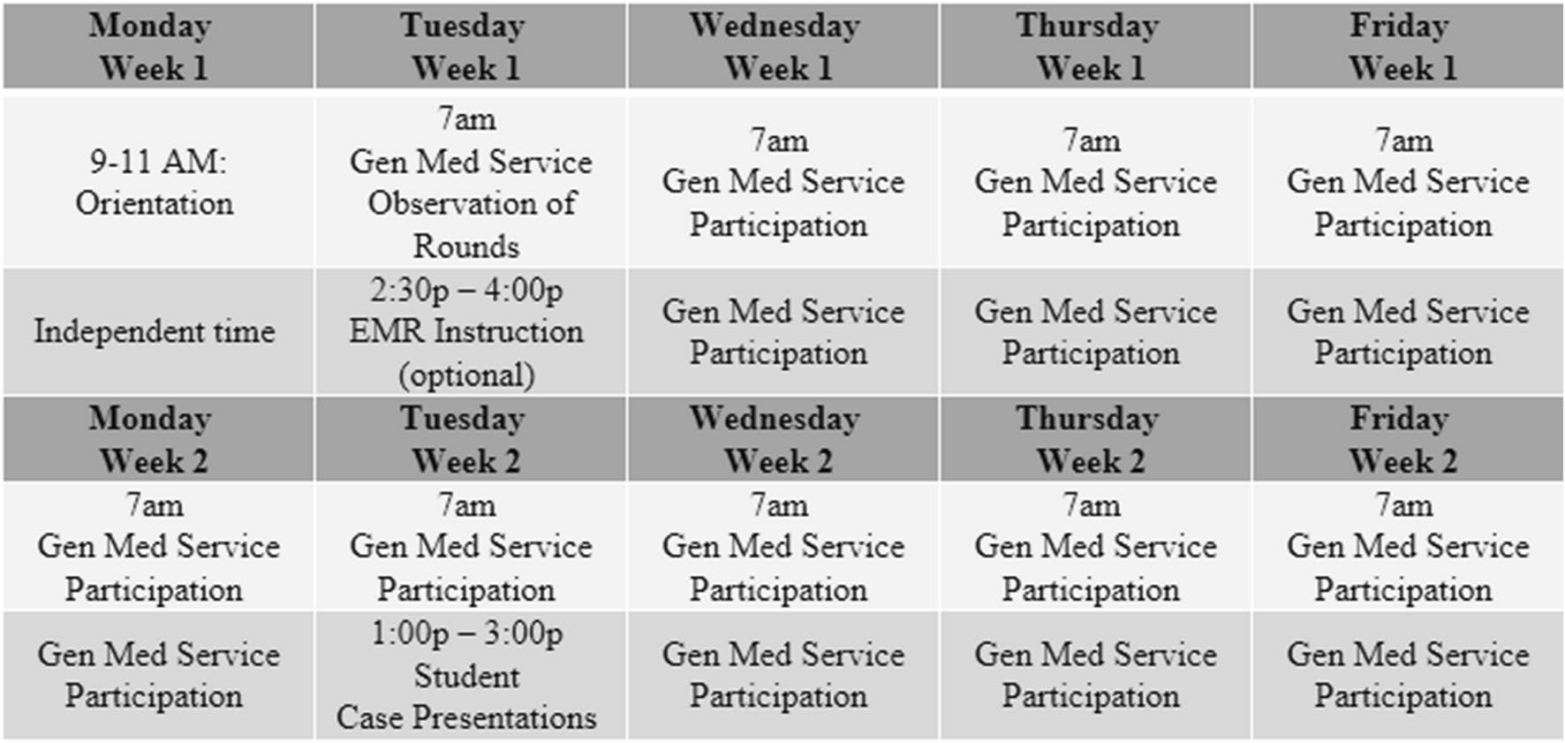
Example schedule schematic

The course was modified over time to include more clinical hours and fewer classroom activities, but a weekly 2-h, faculty-facilitated, small group discussion of clinical cases has been part of the course since inception. In 2022, the course became mandatory for MD-PhD students and in 2023 a 1-week experience supplemented with personalized faculty support was additionally offered. A schematic of the 2024 schedule is seen in Fig. 1.

Student evaluations from 2018 to 2024 were analyzed using descriptive statistics, and two authors thematically analyzed narrative responses. Following IRB exemption, student data were collected from the learning management system, including demographics, LOA reasons, at-risk academic performance indicators, graduation delays after starting the clerkship year, United States Medical Licensing Exam (USMLE) Step 1 Examination performance, and attrition rates. At-risk indicators (“Academic Concern”) included students struggling more than once to demonstrate competency in medical knowledge, patient care, or professionalism in the pre-clerkship years or the clerkship year. Outcomes were analyzed using descriptive and inferential statistics. Students were categorized based on the presence of a LOA before the clerkship year, whether the LOA was planned or unplanned, and participation in RICM. Data from the 2020 cohort were excluded due to the virtual-only format during the COVID-19 pandemic.

Planned LOA students included those with joint degrees or research years, while unplanned LOA students took leave due to USMLE Step 1 examination difficulties, academic challenges, and personal matters. Students in these groups were evaluated for significant differences which could confound results. Categorical variable comparisons between groups were analyzed using the chi-squared (*Χ*^2^) test, with statistical significance defined as *p* < 0.05.

## Results

From 2018 to 2024, 124 students participated in RICM, representing 76% of those who took a LOA before their clerkship year. Among participants, 88 (71%) had a planned LOA, while 36 (29%) had an unplanned LOA. Additionally, there were 40 students who returned from a LOA who did not participate in RICM, of which 60% had an unplanned LOA.

**Fig. 2:**
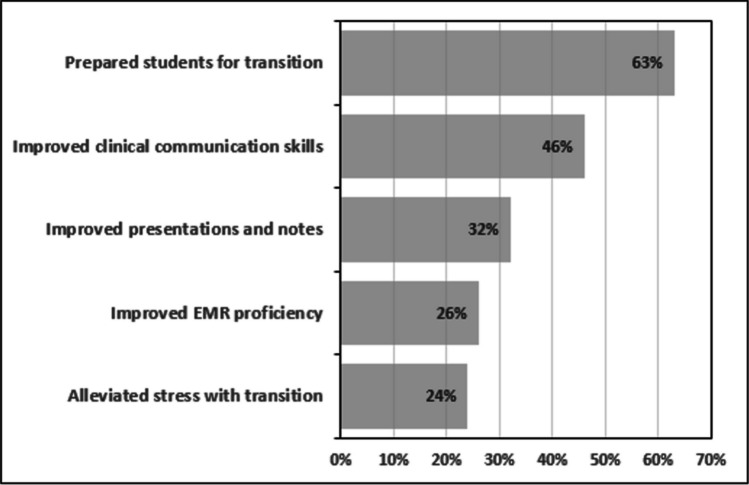
Thematic data from narrative responses for the 5 main themes identified with % respondents who endorsed that theme

The course was evaluated through a post-course survey with an 86% response rate. Students rated the overall course as 4.6 and the wards components as 4.8 on a 5-point Likert scale. Thematic analysis of narrative responses (Fig. 2) identified five major themes from the prompt asking how the course contributed to their learning (Fig. 2). The most common theme was that the course prepared them for their clerkship year (63%), followed by improved communication with patients and the medical team (46%). Notably, 24% of respondents reported that the course helped alleviate stress and anxiety with the transition into the clerkship year.

Table 1 details 4 years of academic data for medical students who began their clerkship year between 2018 and 2022, including those who participated in RICM, those who returned from a LOA but did not participate, and traditional students who did not take a LOA directly before the clerkship year. Data from 2020 were excluded due to the lack of an inpatient ward experience in RICM during the COVID-19 pandemic, and data from 2023 and 2024 RICM students were not yet available at the time of analysis.

**Table 1:** Academic data comparing RICM students with planned and unplanned LOAs, RICM-eligible students who did not participate, and traditional year 3 students without a LOA before entering the clerkship year

	*N* for academic data		Students with academic concern during pre-clerkship years # (%)		Students with academic concern during clerkship year # (%)		Students delayed graduation after starting clerkship year # (%)	
	RICM students	RICM eligible	RICM students	RICM eligible	RICM students	RICM eligible	RICM students	RICM eligible
Planned LOA students	52	14	1 (2%)	1 (7%)	1 (2%) *	3 (21%)	5 (10%)	3 (21%)
Unplanned LOA students	22	12	16 (73%) ***	5 (42%)	9 (41%)	3 (25%)	3 (14%) **	6 (50%)
Traditional year 3 students	712		25 (4%)		20 (3%)		64 (9%)	

*p < 0.01, **p between 0.01 and 0.05, ***p between 0.05 and 0.1. If unmarked *p* > 0.1

The total number of students returning from LOA and included in the academic analysis was 100, representing 12% of all clerkship year students during this period. The majority of students in this sub-analysis returned from a planned LOA and participated in RICM (52%). Demographic analysis revealed that students classified as “under-represented in medicine” accounted for 12% of traditional year 3 students and 33% of LOA students.

Planned LOA students who participated in RICM had academic concerns and delayed graduation profiles similar to traditional year 3 students. However, planned LOA students who did not participate in RICM had more academic struggles and delayed graduations during their clerkship year than planned LOA RICM students. In contrast, unplanned LOA students had more academic concern than any other group, though academic struggles were more common during the pre-clerkship years than the clerkship years (62% vs. 35%, *p* = 0.03). Among students with an unplanned LOA, those who participated in RICM had higher rates of academic concern than non-participants, though this difference was not statistically significant (*p* = 0.35). Unplanned LOA students who participated in RICM had significantly fewer graduation delays than non-participants. Attrition from medical school after starting the clerkship year was rare with only two students out of 812 leaving. Both students had an unplanned LOA, one participated in RICM, one did not.

Among planned LOA students, RICM participants had similar rates of pre-clerkship academic concern compared to eligible non-participants (2% vs. 7%, *p* = 0.65) and lower rates than unplanned LOA students. Among unplanned LOA students, RICM participants had higher rates of pre-clerkship academic concern than eligible non-participants, though this difference was not statistically significant (73% vs. 42%, *p* = 0.07). No significant differences were found in demographic or academic performance variables that would bias the results in favor of RICM. Full data are available in the supplementary material.

## Discussion

The Re-Introduction to Clinical Medicine (RICM) course was designed to support students returning from a leave of absence (LOA) and facilitate their successful reintegration into the medical curriculum. Participants rated the course highly, reporting that it successfully prepared them for their clerkship year. RICM students with planned LOAs, such as for joint degrees or research, had academic outcomes comparable to traditional year 3 students and better than similar students with planned LOAs who did not participate in RICM. Among students with unplanned LOAs, RICM participants had higher rates of academic concern in the clerkship year compared to non-participants, though this difference was not statistically significant. Notably, RICM participants with unplanned LOAs also exhibited higher rates of academic concerns in the pre-clerkship phase, which likely contributed to the observed differences in clerkship performance. RICM participants with unplanned LOAs did experience fewer delayed graduations, a meaningful outcome given the established link between delayed graduation and attrition in this cohort [3]. This study is the first description in the literature of academic outcomes for students returning to the clerkship year after a LOA.

Medical school presents significant stress and mental health challenges, with a meta-analysis showing that 27% of medical students report depression or depressive symptoms, and 11% report suicidal ideation [9]. Additionally, survey data shows depression, burnout, and fatigue are significantly higher among medical students compared to age-matched controls in the general population [10]. Transitioning to the clerkship year has been shown to increase stress for medical students [4] and the limited available data suggests that students returning from a LOA face even greater challenges [5]. Despite these challenges, research on effective support for these students is limited. Findings from the post-RICM survey show the course improves readiness and reduces stress upon the transition from a LOA to the clerkship year, providing valuable insights on this underexamined topic.

With the 2022 transition of The United States Medical Licensing Exam (USMLE) Step 1 to pass-fail and more medical schools adopting pass-fail grading, clerkship evaluations now play a critical role in residency applications. A recent study ranked core clerkship grades as the third most important factor in residency selection [11]. Given these factors, an ungraded preparatory course that enhances reported readiness, communication skills, presentations, and electronic medical record proficiency provides substantial value to both students and medical schools.

Our study has several limitations. The small sample size from a single academic medical center may limit generalizability, though the large size of our medical college and number of years evaluated allows for meaningful analysis and contributes to a gap in published literature on LOA student outcomes during medical school. Additionally, as a retrospective study, causation cannot be established. However, no significant baseline differences were found that would introduce bias in favor of RICM.

## Supplementary Information

Below is the link to the electronic supplementary material.

Supplementary file1 (DOCX 17.8 KB)

## Data Availability

The data that support the findings of this study are openly available at Open Science Framework at https://osf.io/dweqk/files/osfstorage/67a0ce4901ff4e2675b8feeb.
