# Supplementary material for: Navigating the Path Back: A Course for Medical Students Returning from Leave and Their Outcomes
Source: Med Sci Educ. 2025 Mar 4;35(3):1203–7. doi: 10.1007/s40670-025-02345-4 (PMC12228600; doi:10.1007/s40670-025-02345-4)
Supplement: Supplementary file 1 — Supplementary file1 (DOCX 17.8 KB) [file 40670_2025_2345_MOESM1_ESM.docx]

Supplementary Table 1 Full Academic Data

|  | | Planned LOA students | Unplanned LOA students | Traditional Year 3 students |
| --- | --- | --- | --- | --- |
| N for Academic Data | RICM Students | 52 | 22 | 712 |
|  | RICM Eligible | 14 | 12 |  |
| # (%) Students with Academic Concern During Pre-Clerkship Years | RICM Students | 1 (2%) | 16 (73%) *** | 25 (4%) |
|  | RICM Eligible | 1 (7%) | 5 (42%) |  |
| Average USMLE Step 1 Score ^ | RICM Students | 230 | 209 | Not shown |
|  | RICM Eligible | 229 | 209 |  |
| # (%) USMLE Step 1 Failures | RICM Students | 2 (8%) | 7 (32%) | Not shown |
|  | RICM Eligible | 0 (0%) | 3 (25%) |  |
| # (%) Students with Academic Concern During Clerkship Year | RICM Students | 1 (2%) * | 9 (41%) | 20 (3%) |
|  | RICM Eligible | 3 (21%) | 3 (25%) |  |
| # (%) Students Delayed Graduation After Starting Clerkship Year | RICM Students | 5 (10%) | 3 (14%) ** | 64 (9%) |
|  | RICM Eligible | 3 (21%) | 6 (50%) |  |
| # (%) Attrition from Medical School | RICM Students | 0 | 1 (2%) | 0 |
|  | RICM Eligible | 0 | 1 (7%) |  |
| * P < .01, ** P between 0.01 and 0.05, *** P between .05 and 0.1.  if unmarked P> 0.1 | | | | |
| ^ USMLE Score ratings only shown when score is available (2019 and prior).  Not shown for traditional students. | | | | |

Supplementary Table 2 Reasons for Leave of Absence

|  | Planned LOA RICM students | Planned LOA RICM-eligible students | Unplanned LOA RICM students | Unplanned LOA RICM-eligible students |
| --- | --- | --- | --- | --- |
| N for Academic Data | 52 | 14 | 22 | 12 |
| Reasons for LOA | MD-PhD - 36, MD-MPH - 11, MD-JD - 1, MD-MBA - 1, Research - 3 | MD-PhD - 7, MD-MPH - 3, MD-JD - 1, MD-MBA - 1, Research - 2 | Step 1 Failure - 7, Step 1 Delay - 12, other prolonged break - 3 | Step 1 Failure - 3, Step 1 Delay - 9 |
